# Three-Dimensional Positioning for Aircraft Using IoT Devices Equipped with a Fish-Eye Camera

**DOI:** 10.3390/s23229108

**Published:** 2023-11-10

**Authors:** Junichi Mori, Makoto Morinaga, Takumi Asakura, Takenobu Tsuchiya, Ippei Yamamoto, Kentaro Nishino, Shigenori Yokoshima

**Affiliations:** 1 Department of Electrical, Electronics and Information Engineering, Faculty of Engineering, Kanagawa University, 3-27-1 Rokkakubashi, Kanagawa-ku, Yokohama-shi 221-8686, Japan; kenshin@kanagawa-u.ac.jp; 2 School of Engineering, Department of Architecture, Daido University, Nagoya 457-0819, Japan; morinaga@daido-it.ac.jp; 3 Faculty of Science and Technology, Department of Mechanical and Aerospace Engineering, Tokyo University of Science, Noda 278-8510, Japan; t_asakura@rs.tus.ac.jp; 4 Defense Structure Improvement Foundation, Tokyo 160-0003, Japan; d4-yamamoto@bsk-z.or.jp; 5 Kanagawa Environmental Research Center, Hiratsuka 254-0014, Japan; nishino.75n@pref.kanagawa.lg.jp (K.N.); yokoshima.7c7q@pref.kanagawa.lg.jp (S.Y.)

**Keywords:** fish-eye camera, IoT device, MO detection, 3D positioning, aircraft

## Abstract

Radar is an important sensing technology for three-dimensional positioning of aircraft. This method requires detecting the response from the object to the signal transmitted from the antenna, but the accuracy becomes unstable due to effects such as obstruction and reflection from surrounding buildings at low altitudes near the antenna. Accordingly, there is a need for a ground-based positioning method with high accuracy. Among the positioning methods using cameras that have been proposed for this purpose, we have developed a multisite synchronized positioning system using IoT devices equipped with a fish-eye camera, and have been investigating its performance. This report describes the details and calibration experiments for this technology. Also, a case study was performed in which flight paths measured by existing GPS positioning were compared with results from the proposed method. Although the results obtained by each of the methods showed individual characteristics, the three-dimensional coordinates were a good match, showing the effectiveness of the positioning technology proposed in this study.

## 1. Introduction

In the management of aircraft, UAVs and other airborne systems, the measurement of three-dimensional positioning information of aircraft is important, and research and development of various techniques has been carried out. The most popular method is satellite positioning. An Automatic Dependent Surveillance–Broadcast (ADS-B) [1] signal, which is based on GPS technology, is transmitted from almost all civilian aircraft, and the information contained therein is governed by regulations. Furthermore, many airfields are equipped with equipment for radar observation such as Airport Surveillance Radar (ASR) [2], which is used to determine the positions of aircraft that do not transmit an ADS-B signal. The majority of these positioning technologies are based on the principles of 3D position measurement based on detecting the response from an object to a signal transmitted from an antenna. We have regularly been conducting simulations of aircraft noise [3]. For these simulations, it is required to identify the aircraft’s position at relatively lower altitudes below 1000 ft, while measuring frequency characteristics of the aircraft sound. In such a case, if the accuracy of measuring flight altitude is coarse, the precision of the predicted acoustic characteristics related to the flight increases. Therefore, a more accurate technique for estimating flight positions at lower altitude is needed. However, it is difficult to use radar information due to the presence of undetectable aircraft types, the effects of reflections and obstructions from buildings, and issues related to information confidentiality. Furthermore, GPS technologies, including ADSB, are not satisfactory due to the presence of aircraft that do not emit their signals and challenges with quantization errors [4] in GPS altitude measurements. Consequently, an alternative method for estimating flight paths using optical techniques can be effectively adopted.

There has been extensive research on the use of video data captured from the ground for detection of aircraft, and much of this prior work was conducted on themes related to practical application of UAVs in particular. For example, Davies et al. investigated the effectiveness of a Kalman filter for detecting very small aircraft in low-contrast images [5]. Furthermore, Rozantsev et al. successfully detected very small aircraft from images acquired with moving cameras by using computer vision [6]. Doyle et al. developed a system capable of real-time tracking of drones by using a combination of computer vision with pan/tilt technology [7], and Kashiyama et al. have been actively investigating the inference of UAV flight paths by applying cutting-edge technology [8]. These studies have generally investigated detection of aircraft by unique algorithms using video from fixed-focal-point cameras, and we also use these detection methods as a reference. In addition, Kang & Woolsey researched flight path detection by stereoscopic measurement methods using two fish-eye cameras [9]. In contrast, the method in the present paper greatly differs in the sense that it aims to acquire the 3D coordinates of aircraft in accordance with surveying theory. Moreover, the determination of 3D flight paths over a wider range of actual aircraft has not been confirmed in comparison with existing technology, even in case studies. Research into outdoor ground-to-air aircraft detection can therefore be said to have high novelty. Research that captures video of the sky by using a fish-eye camera, unrelated to aircraft, is often found in the field of meteorology. In this research, solar irradiation and other parameters. are inferred from captured sky images [10,11].

We therefore developed an aircraft positioning camera (APC) that is based on low-cost, portable Internet of Things (IoT) devices equipped with a fish-eye camera and that can mechanically measure the three-dimensional position of aircraft in a local region [12]. We are now conducting research to confirm the capabilities of the APC. The remainder of this paper is organized as follows. Section 2 introduces the specifications of the APC, and Section 3 gives the details of the algorithms used for analysis. Section 4 discusses the fundamental calibration experiments, and Section 5 presents a case study for confirming its flight path measurement capabilities. Section 6 includes further discussion including limitations, and finally, Section 7 concludes the paper.

## 2. IoT Device Mounted with Fish-Eye Camera

**Figure 1:**
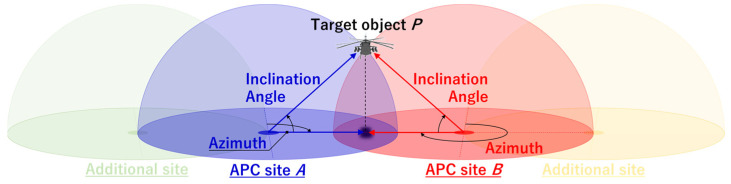
Flight aircraft positioning method by our method using APCs.

Figure 1 shows an overview of the measurement method used by the APC for obtaining the 3D positions of aircraft. At sites A (blue) and B (red) in the diagram, the dome shapes extending into the sky centered on each measurement point indicate the limits of the fish-eye cameras. This method assumes that APCs are installed at no less than two sites, and that aircraft passing through the sky are captured simultaneously by each camera. Although the distance between the sites cannot be rigorously specified because it depends on the number of pixels in the installed cameras, around 300 m is thought to be the tolerable limit empirically based on the performance of the system in this study as described below.

**Figure 2:**
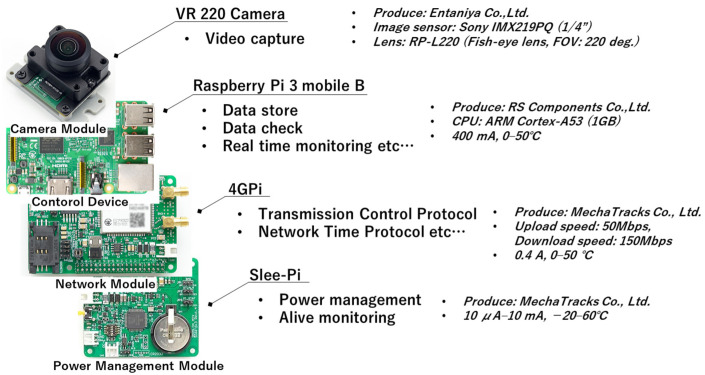
Construction of APC based on a Raspberry Pi 3.

Figure 2 shows a diagram of the components of the APC hardware, and Table 1 shows the specifications of each part. A single-board computer (Raspberry Pi 3 model B) was used as the system board that controlled the video capture, encoding, Transmission Control Protocol (TCP) communication via a network module (4GPi), time management and power supply management via a power supply unit (sleePi). A dedicated camera module for Raspberry Pi (VR220) was used as the camera tool, and this was mounted with a fish-eye lens (RP-L220) for equidistant shooting that can capture a field of view of up to a maximum of 220°. Because sudden rain was also anticipated at the actual shooting site, this equipment was protected with a custom-made waterproof case. Although it is desirable to measure the tilt and level azimuth of the APC continuously and accurately, for example, by using a tilt sensor or geomagnetic sensor, in this study these were simply adjusted by using an electronic compass and water level. In this research, the output pixel size of the video was set to 960 pixels in both width and height to save on the capacity of measured data.

## 3. Algorithm for Estimating Flight Path

**Figure 3:**
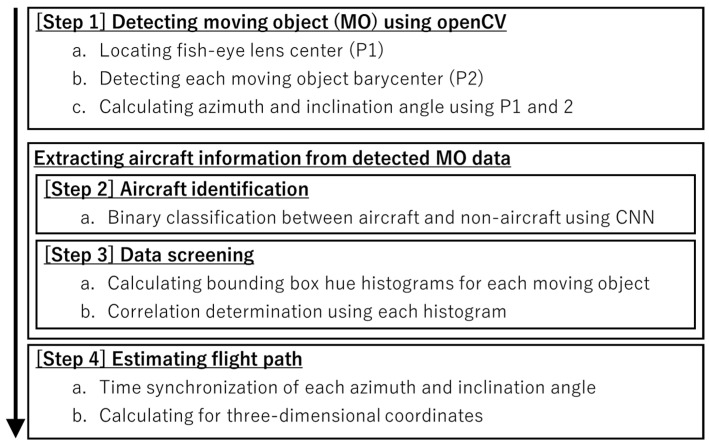
Algorithm flow chart for flight path estimation.

Figure 3 shows a flowchart of the analysis algorithm for flight path inference in this study. Methods that employ YOLO [13] for this kind of moving object (MO) identification have become mainstream in recent years, and our system can potentially also be migrated to this method in the future. As shown in Figure 3, a conventional method based on MO detection and a convolutional neural network (CNN) [14] was used to perform post facto analysis of the video captured at each site. First, MO detection of barycenter pixels of all MOs captured in the video at each site was performed using openCV [15], and the values of the azimuth angle and angle of elevation of the MO at each time were calculated from the coordinates and center of the fish-eye lens (Step 1). Although the sky occupies most of the image when the sky is filmed using a fish-eye lens, the ground is also captured around the edges of the lens because the field of view is wide. Because of this, the MOs from MO detection are not limited to flying objects such as aircraft, birds and insects. MOs are also detected on the ground, such as people, cars and trees, and these constitute noise that obscures the information about the aircraft. To extract aircraft information from the data, aircraft identification is performed by CNN (Step 2), and processing combines this with data screening based on this identification result (Step 3). In Step 4, the extracted continuous values of the angle of each aircraft at each site are time synchronized, and the coordinates are calculated by the method of forward intersection from surveying [16] based on this angle information and the coordinates of each measurement site. The details of the processing method in each step are described below.

### 3.1. MO Detection Using openCV (Step 1)

**Figure 4:**
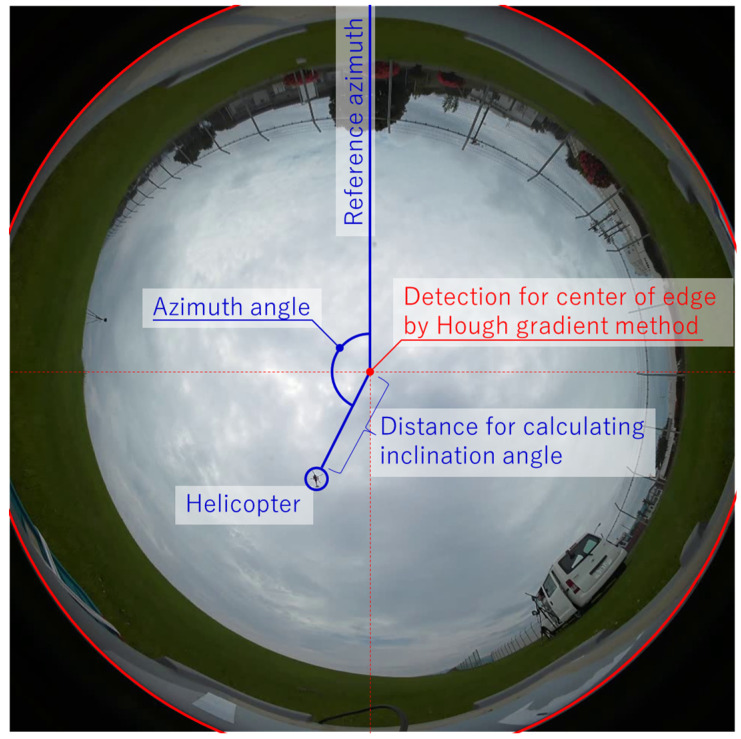
Image of the calculation of azimuth and inclination angles in a fish-eye camera image.

**Figure 5:**
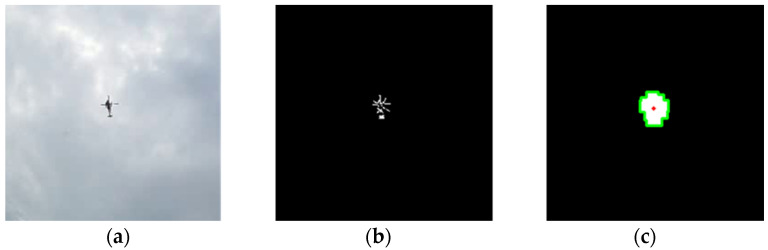
Example frames for calculations in motion detection analysis: (**a**) original video; (**b**) result of frame difference calculation, grayscale conversion and image binarization; (**c**) result of dilation, object contour detection and barycentric coordinate estimation.

Figure 4 shows an image of the calculation of the azimuth angle and angle of inclination from the fish-eye camera images, and Figure 5 shows example frames of each analysis step in MO detection. This analysis method is based on the idea of inferring the inclination angle from the distance between the center coordinate of the circle of the fish-eye lens and the barycenter coordinates of the MO, and the azimuth angle from the angle formed by these two coordinates and the reference azimuth (in this study, the upward direction in the image of the system). The center coordinate is detected by Hough transformation of the center of the fish-eye lens captured in the image (red lines in Figure 4). The barycenter coordinate is calculated by using a simple MO detection technique. More specifically, the difference between consecutive frames is calculated by the frame difference method, and the difference image is converted to grayscale and then binarized (middle panel in Figure 5). This binarized image is composed of a set of many minuscule points (white is MO, black is not MO), and the white parts must be joined to be recognized as a region. Because of this, the discontinuous white regions are joined by applying the values of the white pixels to the surrounding black regions by expansion processing. The silhouette of the MO region is detected from the image, and finally the pixel coordinates of the barycenter of that silhouette are defined as the position of the MO (right panel in Figure 5).

The azimuth angle θ and inclination angle φ are calculated by the following equations based on the center (*F_x_*, *F_y_*) of the fish-eye lens as calculated by the above method and the barycenter coordinates (*O_x_*, *O_y_*) of the MO:

(1) $$\theta =\arctan\left(F_{x}-O_{x},F_{y}-O_{y}\right),$$

(2) $$\varphi =\sqrt{{\left(F_{x}-O_{x}\right)}^{2}+{\left(F_{y}-O_{y}\right)}^{2}}/p_{\mathrm{ave}},$$

where *p*_ave_ is a ratio obtained from calibration experiments described below for converting the distance between pixels into an angle.

### 3.2. Extracting Aircraft Information from Detected MO Data

**Figure 6:**
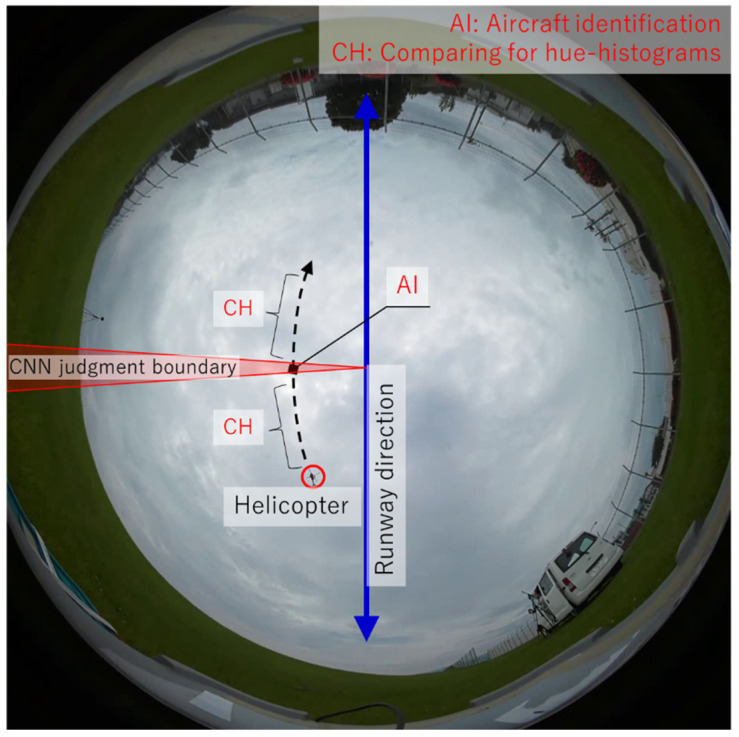
Analysis image for extraction of aircraft information from detected MO data.

Figure 6 shows an image for the extraction of aircraft information from data calculated by the above MO detection. In cases such as urban areas and green areas where the video captured by the APC can easily capture MO other than aircraft, the amount of MO information analyzed in Step 1 increases greatly. When mechanical processing is performed on this kind of MO data, the processing time also increases proportionally. In this study, the measurement target is aircraft flying at low altitude near airfields, and these aircraft regularly fly along a path in the runway direction (blue line in Figure 6). In this study, boundary lines (red lines in Figure 6) through which the aircraft always pass were set on the camera lens, and a CNN was used to perform binary classification limited to MOs that pass through this region. The classification of aircraft outside this range and other MOs is determined by calculating the correlations of the hue histograms for each MO at each moment.

**Figure 7:**
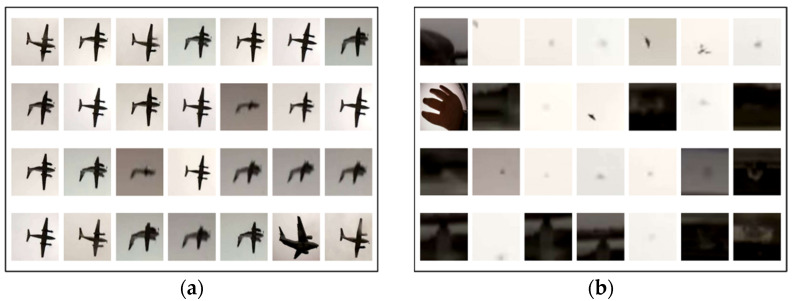
Example images from the dataset for training of the CNN: (**a**) aircraft such as jet planes, prop planes and helicopters; (**b**) non-aircraft such as humans, birds, flying insects, trees and cars.

In the binary classification of aircraft and other MOs using a CNN, bounding box images of the MO detected at the same time as the MO detection calculation are used as training data. Figure 7 shows an example image of a training dataset. For the video for obtaining the training data, data captured in advance by the APC on a different day in the experiment were used in the case study described below. For classification annotations, all images are classified by visual determination as “aircraft” or “other” in the analyzed bounding box images. The aircraft set (left side in Figure 7) contains multiple aircraft types including jet planes, propeller planes and helicopters, and the non-aircraft set (right side in Figure 7) contains MOs such as people, birds, insects, trees and vehicles. In each category, 1000 images were used for training.

Table 2 shows the setting items for training the CNN. In this study, PyTorch [17] was used as the framework, and after the ResNet-18 structure [18] and weightings of the trained model were loaded by following the tutorial, it was trained using the above dataset. Parameters such as loss coefficients and activation coefficients were set to default values.

To investigate the performance of binary classification using this CNN, classification was performed on 3200 unknown measured images that were not contained in the training data (1600 in each category). Table 3 shows the confusion matrix of the results. The vertical direction shows the prediction results and the horizontal direction shows the classification results. The overall accuracy as shown at the bottom right was 86.7%. However, if we look at the aircraft column for the predicted label, this model had an accuracy of 99.9% for aircraft prediction, with almost no misidentifications. This high accuracy was obtained because the MO is clear when a low-altitude flying MO near a runway is captured by the APC, as shown in Figure 7. These conditions make it easy to distinguish aircraft MOs from non-aircraft MOs. The subsequent data screening was performed based on the judgment results from this model.

### 3.3. Data Screening (Step 3)

**Figure 8:**
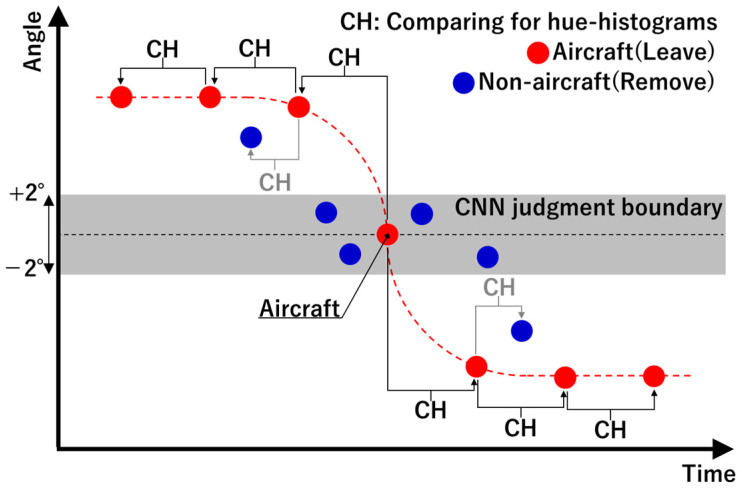
Image of data screening by comparison of hue histograms of MO bounding box images based on CNN judgment. The vertical axis is angle, and the horizontal axis is time. Red plots represent aircraft and blue plots represent other MO information.

Figure 8 shows an image of the data screening based on judgment results from the CNN. First, a range of ±2° was set as the CNN judgment boundary, and binary classification by the CNN was performed on the bounding box images of MOs passing through this region. In this way, hue histograms were obtained for the bounding box images of MOs judged to be aircraft in the range of the boundaries, and the correlation coefficient was calculated between temporally adjacent MOs. If this correlation coefficient exceeds a threshold, the MO is judged to be an aircraft; otherwise, it is a non-aircraft. Then, screening is performed on only the aircraft information from among the MO information in the entire region by repeatedly performing this processing over time while updating the original histogram information. In this study, a correlation coefficient of ≥0.85 and a time interval of ≤2 s were set as the threshold for the correlation coefficient for judgment.

**Figure 9:**
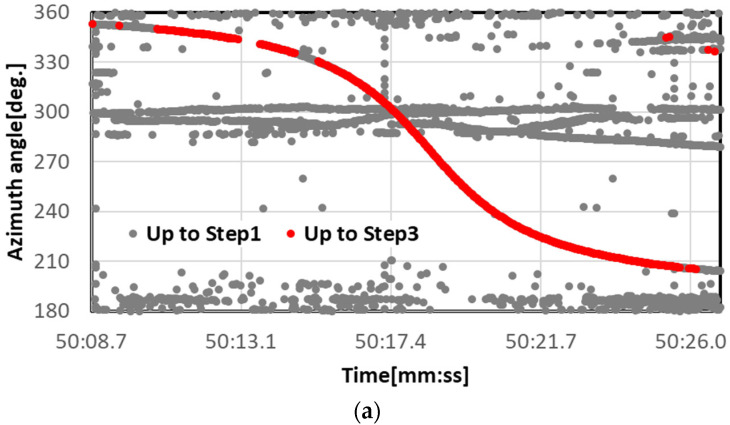

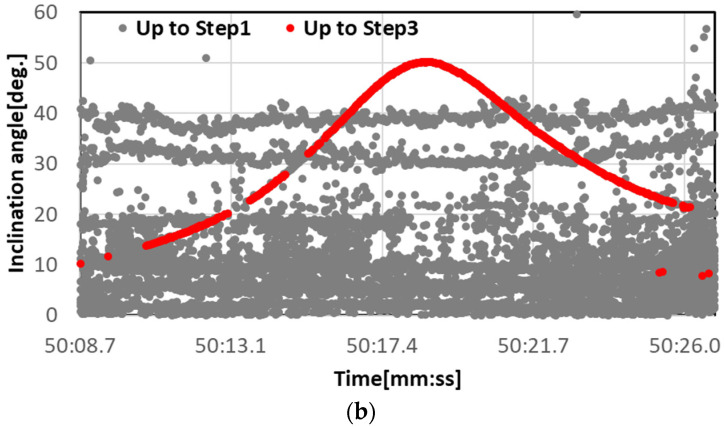
Example results of extracting aircraft information obtained from Steps 1 to 3. The vertical axis in (**a**) is the azimuth angle and that in (**b**) is the inclination angle. Both horizontal axes are time. The gray plots show the results up to Step 1, and the red plots show the results up to Step 3.

Figure 9 shows an example of the results of extracting the aircraft information by using Steps 2 and 3. In this example, although there were several outliers at low inclination angles in the right side of the figure, extremely clean angle variation properties appeared outside this angle range. The outliers at low inclination angle are thought to be due to the effect of the increasing inclusion of non-aircraft MOs in the hue histogram judgment.

### 3.4. Estimation of Flight Position

If the time-continuous values of azimuth angle (*θ_A_*, *θ_B_*) and inclination angle (*φ_A_*, *φ_B_*) up to Step 3 can be extracted at site A (*A_x_*, *A_y_*, *A_z_*) and site B (*B_x_*, *B_y_*, *B_z_*) around a flying aircraft *P* as shown in Figure 1, then the three-dimensional flying position (*P_x_*, *P_y_*, *P_z_*) of the aircraft can be calculated using the following equations:

(3) $$P_{x}=\frac{m_{2}\cdot B_{x}-m_{1}\cdot A_{x}+A_{y}-B_{y}}{m_{2}-m_{1}},$$

(4) $$P_{y}=m_{1}\cdot \left(P_{x}-A_{x}\right)+A_{y},$$

(5) $$P_{z}=A_{Z}+\left(D\cdot m_{3}\right),$$

where $m_{1}=tan\theta_{A}$, $m_{2}=tan\theta_{B}$, $m_{3}=tan\varphi_{A}$, and D is the horizontal distance between site A and the aircraft. Note that since *m*_1_ and *m*_2_ diverge due to the *tan* calculation when the azimuth angle at both sites is 90° or 270°, the following measure to prevent this was taken in this study:

(6) $$P_{x}=\frac{m_{2}\cdot B_{x}-m_{1}\cdot A_{x}+A_{y}-B_{y}}{m_{2}-m_{1}}$$

(7) $$P_{y}=m_{1}\cdot \left(P_{x}-A_{x}\right)+A_{y}$$

To perform the above calculations, it is necessary to synchronize the azimuth and inclination angles obtained at each location of Sites A and B. In this study, these data were synchronized by using the Network Time Protocol time information measured at each site.

## 4. Calibration Tests for Fish-Eye Lens

Calibration experiments were conducted to acquire the *p*_ave_ for calculating the inclination angle and to confirm the amount of distortion of the fish-eye lens used. In this experiment, angle markings on the walls and ceiling were captured by the APC, and the pixel distance between the markings and the ceiling center point of the fish-eye camera was determined in the image. The details of this experiment are shown below.

### 4.1. Conditions for Test

**Figure 10:**
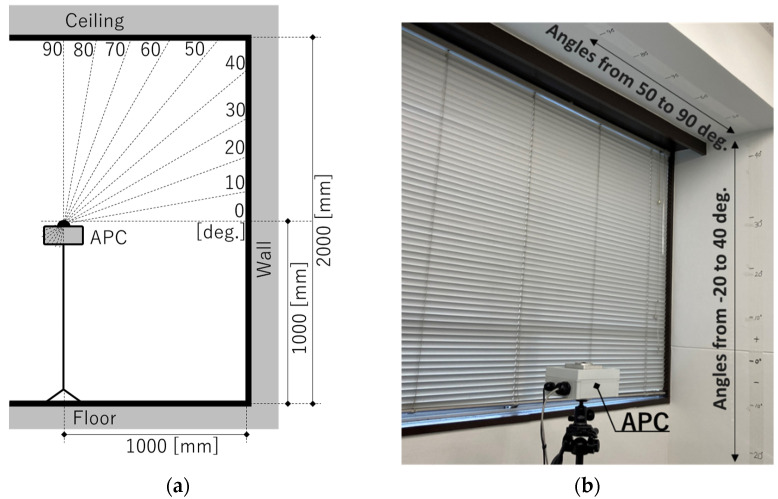
Calibration test image and photography: (**a**) schematic of the experiment; (**b**) actual photograph.

Figure 10 shows a schematic of the experiment and an actual photograph. The experiments were performed in the corner of a meeting room. The field of view of the fish-eye lens being used was 220°, and precise positions of 12 locations at intervals of 10° from −20° to +90° on the wall and ceiling were adjusted by laser rangefinder to cover half of that inclination angle range (110°). Note that in this investigation, the base of the fish-eye lens was assumed to be 0°. The APC was mounted on a tripod with a camera platform marked at 30° pitch, and 6 images were captured sequentially while rotating the camera orientation in 60° increments relative to the wall.

### 4.2. Results

**Figure 11:**
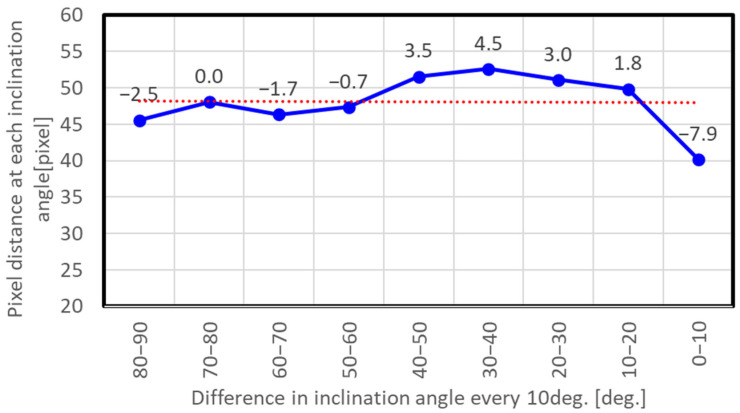
Calibration test result for an APC. The horizontal axis is the difference in inclination angles every 10°, and the vertical axis is the pixel distance at each inclination angle. The solid line is the arithmetic mean of trials in all directions for the APC, the dotted line is its linear regression line, and the data labels are the difference from the arithmetic mean of pixel distances at each inclination angle.

Figure 11 was obtained by taking the pixel coordinates of the center of the dome (90°) as the starting point and then lowering the starting point sequentially in 10° increments at the pixel distance between the coordinates at an angle 10° lower. The solid line shows the arithmetic mean over all trials when the angle of the APC to the wall was changed six times, the dotted line shows the arithmetic mean of the pixel distances obtained at all inclination angles, and the labels on each dot show the difference from the dotted line. Since the deviation depending on camera angle was less than one pixel, only the results for the arithmetic mean are shown. The arithmetic mean for all inclination angles was 48.07 pixels. Note that variation in value was large between different inclination angles, and particularly that from 10° to 0°. Furthermore, the arithmetic mean for the angles above 50° was relatively small, and the arithmetic mean for the lower angles excluding from 10° to 0° was relatively large. The fish-eye lens mounted on the equipment ideally offers equidistant shooting, but it actually had the distortion as shown in the results. We therefore adopted 4.807 pixel/degree as derived from the mean pixel distance of 48.07 pixels above as the *p*_ave_, and also added the difference values at each inclination angle as determined from the experiments as correction terms for each calculated angle.

## 5. Case Study

We conducted a case study on determining the three-dimensional flight path of aircraft passing overhead for landing by using an APC at Fureainomori Park at the Atsugi Airfield in Kanagawa Prefecture, Japan, in May 2022. In this case study, simultaneous monitoring by ADS-B was performed and the ADS-B flight path was compared with that from the APC in order to assess the accuracy of the flight path obtained by the APC. The details of this experiment are described below.

### 5.1. Conditions

**Figure 12:**
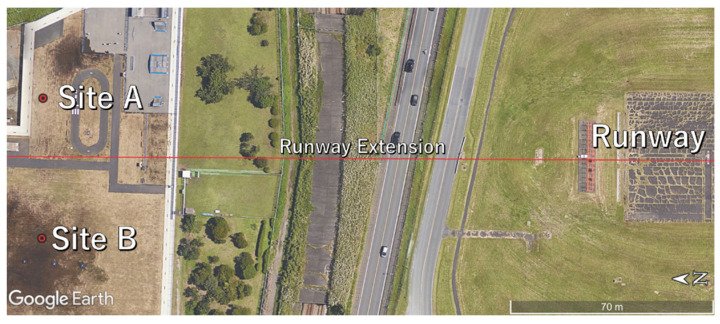
Measuring sites at Atsugi Airfield.

**Figure 13:**
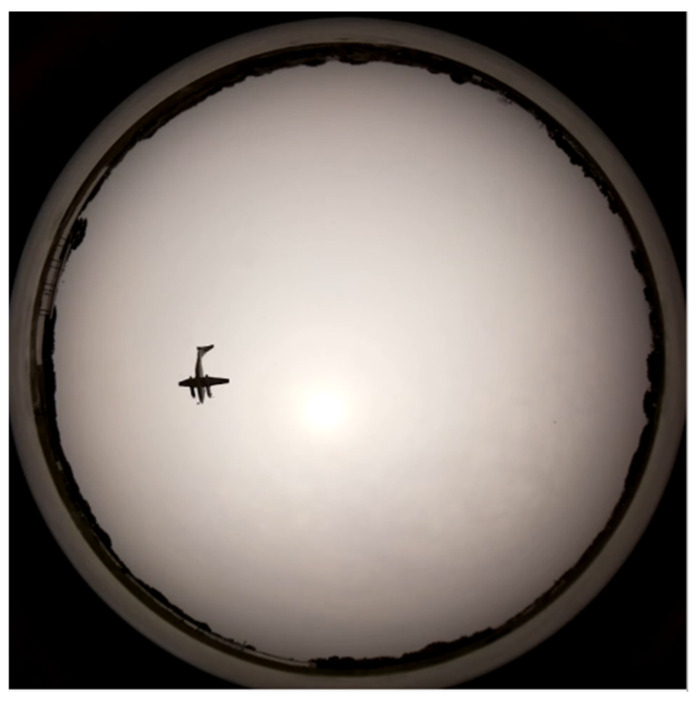
Sample frames of video taken by the APC under cloudy conditions.

Figure 12 shows the measurement sites. This park is located about 150 m from the northern end of the runway, and aircraft pass overhead just before landing. The predicted flight paths of approaching aircraft were provisionally calculated based on the touchdown point and approach angle (assuming a gradient of 3°), and positions that give an inclination angle of 60° were adjusted with respect to the predicted flight paths. Figure 13 shows a sample frame of video captured during measurements. The weather on the measurement day had thin clouds that spread across the sky evenly without breaks, and the sun was also faintly visible. The flight paths were calculated by APC through the analysis method described above based on the video obtained through this measurement. The flight path obtained by the APC and the flight path obtained by ADS-B were converted into XYZ coordinates centered on the runway and cleaned up.

### 5.2. Results and Discussion

**Figure 14:**
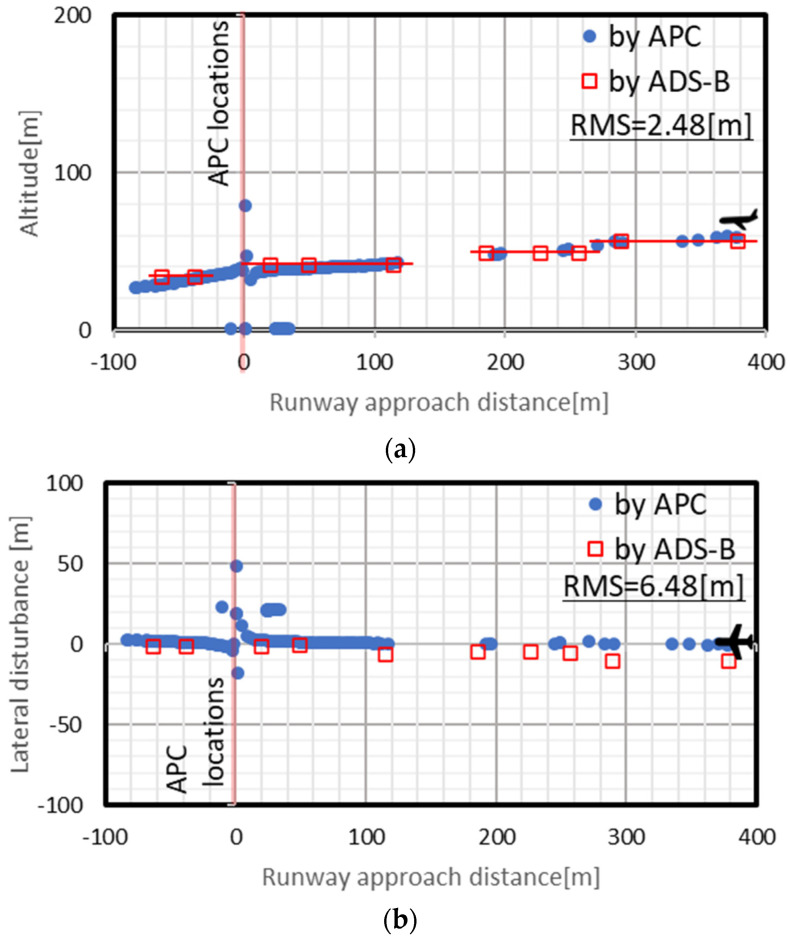
Comparison between flight paths by APC measurement and ADS-B monitoring: (**a**) vertical profile; (**b**) horizontal plane. In each graph, the circle plots are the APC path, the square plots are the ADS-B path, the vertical solid line on the left side of each graph is the location where the APC was installed, and the landing direction is toward the left in each panel. The root mean square errors between the close plots obtained by the two methods are indicated in each panel as RMS.

Figure 14 shows the flight paths obtained by both methods. In both graphs, the circles show the APC path and the squares show the ADS-B path, and the solid line on the left side shows the position where the APC was installed, and the landing direction of the aircraft is toward the left side of each panel. The root mean square errors between the close plots obtained by the two methods are indicated in each of the figures as RMSs.

The APC path had scatter perpendicular to the direction of motion at the camera installation positions. Furthermore, the plots are more scattered with increasing distance from the camera installation position. In comparison, the ADS-B path was more scattered than the APC path overall. For the ADS-B altitudes in particular, a stepped shape with intervals of approximately 7.7 m was measured.

The scatter in the APC path perpendicular to the direction of motion is thought to be because the values diverged to infinity when the *tan* component of the azimuth angle approached 90° or 270°. In addition, the cause of scatter in the APC path in the area far from the camera installation location is attributed to the MO captured in the video being small, and the MO detection was interrupted by the cloud conditions in the sky. The reason why the ADS-B path was more scattered than the APC path is thought to be that the ADS-B transmission interval was around 0.5 s [19] according to the specifications, whereas for the camera the time resolution was 30 fps (an interval of around 0.03 s). In particular, since ADS-B altitude is barometric altitude or GNSS geometric altitude, a quantization error of 25 ft (≈7.7 m) occurs and that quantized performance was evident in the results. Although the paths for each method had various characteristics, they had almost identical 3D trajectories.

## 6. Discussion and Limitations

This study showed that APC can infer the local path of a flying aircraft, similar to existing technology, but many issues remain that need to be investigated. We are planning further research themes using this system. The details are described below.

The issues that require investigation include (1) the policy for setting each of the parameters in MO detection; (2) the effects of errors during installation; (3) the detection limit; (4) comparisons with previous research; and (5) investigation of the effects of weather and other factors.

For issue 1, the first parameters are the setting values for MO detection in openCV in the APC in this study, which were adjusted empirically in this work. These parameters will need to be changed depending on the equipment used, the types of MO targeted, and effects of factors such as the video capture environment. As a result, the policy for sophisticated setting of the parameters under various conditions needs to be investigated.

Regarding issue 2, error during installation included misalignment when the APC was installed at the site, with parameters such as the tilt adjusted manually in this study. The size of the effect that this installation error has on the output results needs to be confirmed. Depending on the situation, a mechanism for digital error correction also needs to be considered.

With respect to issue 3, the detection limit is the range that can be captured by the APC, and in the results of the case study in this report, detection was within a range of around 500 m. However, we envision that this range will vary depending on conditions such as the type of aircraft, contrast with the background, and speed of the aircraft. As a result, it is desirable to accurately investigate the measurement thresholds for MO detection under various flight parameters.

To address issue 4, comparisons should be made with the previous research listed in Section 1. For a given type of airborne MO, it is desirable to rigorously confirm the differences in performance between the cases of using fixed-focus-point cameras, fish-eye lens stereo cameras and the present APC.

For issue 5, the weather conditions during video capture need to be considered. In the present case study, the path in Figure 14 was obtained under cloudy conditions as shown in Figure 13. However, there is a high possibility that tracking by camera is not possible in cases including precipitation, low thick clouds, bright sunlight and nighttime. Knowing these detection limits is also important in terms of metrology, and some method that enables the inference of paths during bad weather by digital processing would also be desirable to investigate.

Further research themes that are planned are as follows: (1) dynamic laboratory experiments employing computer graphics and three-dimensional video capture technology; (2) the ability to measure source emission power; and (3) applications such as surveys counting the number of flights.

The laboratory experiments in research theme 1 are envisioned to target markers that are actually moving, unlike the static calibration experiments shown in Section 4. In the envisioned experiment, computer graphic video with an arbitrarily created moving MO will be projected onto a 3D dome by employing computer graphics and 3D projection technology, and the MO will be captured on video by the camera. If such a virtual experimental method is effective, it will make it possible to investigate issues 1 to 4 as described earlier, and to conduct rigorous investigation by changing the size, speed and contrast with the background of the target MOs. This would also allow investigation of misalignment during installation and the effect of the type of camera.

Measurement of source emission power in research theme 2 means simultaneously measuring the acoustic power of noise emitted at every instant at the position of the aircraft, and this information will become important data for preparing noise maps for evaluating the impacts of aircraft noise. A method of noise measurement based on mutual correlation methods employing multichannel microphones [20] is conventionally used but requires large equipment and high costs. If the same measurement can be performed with the APC, it would have the advantages not only of convenience, but also the ability to easily identify the types of aircraft from video, in contrast to sound measurements.

Surveys counting the number of flights in research theme 3 above would entail measurement of operating performance such as active times of aircraft arriving at and departing from an airfield, operational directions, flight modes and flight paths, and these data are considered important when preparing noise maps. Since the majority of civilian aircraft transmit the ADS-B signal, it is easy to determine their total number of flights. However, military aircraft often do not transmit this signal, so technology for observing the number of active aircraft is needed in addition to ADS-B. It is thought that the APC can be applied effectively as one of these methods.

## 7. Conclusions

In the management of aircraft, UAVs and other airborne systems, measurement of three-dimensional position information of aircraft is important. Against a backdrop where research and development of various technologies has been carried out, we developed an APC that is based on IoT devices equipped with a low-cost, portable fish-eye camera and can mechanically measure the three-dimensional position of aircraft in a local region. This paper introduced the specifications of this APC, the details of the algorithms used for analysis, fundamental static calibration experiments and a case study for confirming its flight path measurement capabilities. The three-dimensional flight paths of aircraft in the situations of takeoff and landing were measured by the two methods of APC and ADS-B. Although the paths obtained by each of the methods had individual characteristics, the RMS values of the three-dimensional coordinates measured by the present method indicated only a minor discrepancy of 2.48 m for the vertical profile and 6.48 m for the horizontal plane. This fact indicates that the APC method enables detailed position measurements at relatively lower altitudes, which was difficult to measure with existing technologies such as radar and ADS-B.

By applying the technology of N-view triangulation [21], the APC method presented in this study has the potential to increase the measurement accuracy. In future work, the current method can be expanded to become more accurate by increasing the number of reference points.

## Figures and Tables

**Table 1:** Specifications for the sensing camera system.

Parts	Product	Manufacturer	Purpose
Single-board computer	Raspberry Pi 3 mobile-B	---	Video filming Data encoding Control
Network module	4GLTEpi	MechaTracks Co., Ltd., Fukuoka, Japan	TCP Time control
Power management unit	sleePi	MechaTracks Co., Ltd., Fukuoka, Japan	Supply voltage, alive monitoring
Camera module	VR220	Entaniya Co., Ltd., Tokyo, Japan	---
Fish-eye lens (Equidistant projection)	RP-L220	Entaniya Co., Ltd., Tokyo, Japan	---
Waterproof case	---	MechaTracks Co., Ltd., Fukuoka, Japan	---

**Table 2:** Setting items for training of the CNN.

Setting Items	Configuration	Version
GPU	NVIDIA GeForce RTX	3070 Laptop
OS	Ubuntu	20.04
Language	Python	3.8.10
Framework	Pytorch	1.12.0 + cu113
Pre-trained model	ResNet	18

**Table 3:** Binary classification accuracy between aircraft and non-aircraft based on CNN. *N* is the number of images in a category, and the percentages are each classification accuracy. “Total” in the bottom right is the overall model accuracy.

	Aircraft	Non-Aircraft	*N*	Recall [%]
**Aircraft**	1176	424	1600	73.5
**Non-aircraft**	1	1599	1600	99.9
***N***	1177	2023	3200	
**Precision [%]**	99.9	79.9	Total	86.7

## Data Availability

The data that support the findings of this study are available from the corresponding author, J.M., upon reasonable request.

## References

[1] Strohmeier M., Lenders V., Martinovic I. (2015). On the security of the automatic dependent surveillance-broadcast protocol. IEEE Commun. Surv. Tutor..

[2] Taylor J.W., Brunins G. (1985). Design of a new airport surveillance radar (ASR-9). Proc. IEEE.

[3] Bui T.L., Nguyen T.L., Morinaga M., Morihara T., Hiraguri Y. (2021). Effect of measurement-based noise source model of military airplanes on the validity of aircraft noise estimation in Vietnam. Acoust. Sci. Technol..

[4] Falk C., Gonzales J., Perez J. Using Automatic Dependent Surveillance-Broadcast Data for Monitoring Aircraft Altimetry System Error. Proceedings of the AIAA Guidance, Navigation, and Control Conference.

[5] Davies D., Palmer P., Mirmehdi M. Detection and Tracking of Very Small Low Contrast Objects. Proceedings of the British Machine Vision Conference.

[6] Rozantsev A., Sinha S.N., Dey D., Fua P. Flight Dynamics-based Recovery of a UAV Trajectory using Ground Cameras. Proceedings of the IEEE Conference on Computer Vision and Pattern Recognition.

[7] Doyle D.D., Jennings A.L., Black J.T. (2014). Optical flow background estimation for real-time pan/tilt camera object tracking. Measurement.

[8] Kashiyama T., Sobue H., Sekimoto Y. (2020). Sky Monitoring System for Flying Object Detection Using 4K Resolution Camera. Sensors.

[9] Kang C., Woolsey C.A. Optimal Placement Algorithm for Multiple Heterogeneous Stereo Vision Systems. Proceedings of the AIAA AVIATION Forum.

[10] Chapman L., Thornes J. (2004). Real-Time Sky-View Factor Calculation and Approximation. J. Atmos. Ocean. Technol..

[11] Gil G.G., Ramirez J.M. (2019). Fish-eye camera and image processing for commanding a solar tracker. Heliyon.

[12] Mori L., Morinaga M., Yamamoto I., Yokota T., Makino K., Hiraguri Y. (2019). Development of aircraft tracking camera system for sound power level measurement of aircraft noise. INTER-NOISE and NOISE-CON Congress and Conference Proceedings.

[13] Jiang P., Ergu D., Liu F., Cai Y., Ma B. (2022). A Review of Yolo Algorithm Developments. Procedia Comput. Sci..

[14] Xie L., Yuille A. Genetic CNN. Proceedings of the IEEE International Conference on Computer Vision (ICCV).

[15] Howse J. (2013). OpenCV Computer Vision with Python.

[16] Cheng X., Li Q., Zhou W., Zhou Z. (2020). External Deformation Monitoring and Improved Partial Least Squares Data Analysis Methods of High Core Rock-Fill Dam (HCRFD). Sensors.

[17] Kochgaven C., Mishra P., Shitole S. Detecting Presence of COVID-19 with ResNet-18 using PyTorch. Proceedings of the International Conference on Communication information and Computing Technology (ICCICT).

[18] He K., Zhang X., Ren S., Sun J. Deep Residual Learning for Image Recognition. Proceedings of the 2016 IEEE Conference on Computer Vision and Pattern Recognition (CVPR).

[19] Pryt R.V.D., Vincent R. (2015). A Simulation of the Reception of Automatic Dependent Surveillance-Broadcast Signals in Low Earth Orbit. Int. J. Navig. Obs..

[20] Yamada I., Hayashi N. (1992). Improvement of the performance of cross correlation method for identifying aircraft noise with pre-whitening of signals. J. Acoust. Soc. Jpn. (E).

[21] Salguero M.G., Jimenez J.G. (2023). Certifiable Solver for Real-Time N-View Triangulation. IEEE Robot. Autom. Lett..

